# Environmental noise exposure in schools in São Paulo, Brazil: potential noise sources and health impacts among teachers

**DOI:** 10.1038/s41598-026-45322-6

**Published:** 2026-03-26

**Authors:** Camila Quintiliano de Andrade, Natalia Vincens, Adelaide Cassia Nardocci, Clayton Henrique Rocha, Martin Röosli, Alessandra Giannella Samelli

**Affiliations:** 1 Department of Physical Therapy, Speech-Language-Hearing Pathology Sciences, and Occupational Therapy, Faculty of Medicine (FMUSP), University of São Paulo, São Paulo, Brazil; 2 Sound Environment and Health, School of Public Health and Community Medicine, Institute of Medicine, University of Gothenburg, Box 453, Gothenburg, 40530 Sweden; 3 Faculty of Public Health, University of São Paulo, São Paulo, Brazil; 4 Swiss Tropical and Public Health Institute, Allschwil, Switzerland; 5 University of Basel, Basel, Switzerland

**Keywords:** Environmental noise, School environment, Teachers’ health, Noise annoyance, Noise sensitivity, Sleep quality, São Paulo, Occupational exposure, Environmental sciences, Environmental social sciences, Health care, Risk factors

## Abstract

Noise is an important environmental and occupational health risk factor linked to sustainable development. Schools are key environments where teachers may face harmful noise exposure, yet data from low- and middle-income countries remain limited. This study assessed school noise levels and related health impacts among teachers in western São Paulo. Using a short-term repeated-measures design, daily noise measurements were conducted over one school week in seven schools, complemented by cross-sectional multilevel analyses of teacher-reported outcomes. Continuous environmental noise data were collected with calibrated Type-II sound level meters positioned at the most exposed facades, and 85 teachers completed validated questionnaires on noise sensitivity, annoyance, self-rated health, wellbeing, and sleep quality. Median weekday noise exposure reached 70.3 dB LAeq(7–19) and 94.2 dB LAFmax(7–19). Higher student density and nearby commercial activity were associated with increased noise levels. Teachers reported substantial annoyance from children’s irrelevant conversations/screams (48%), road traffic (35%), and neighboring schools (31%), and more than 60% were highly noise-sensitive at work. A 10 dB increase in LAeq(7–19) was associated with 4.2 times higher odds of poor self-rated health (OR = 4.2, 95% CI: 1.3–14.0) and significantly higher noise sensitivity scores. Teacher’s sleep quality and wellbeing also varied across schools with differing noise levels. Although exposure to noise in the school environment represents contextual environmental conditions and not an individual dose in indoor settings, the results suggest that teachers are exposed to environmental noise above recommended limits, with measurable health impacts. Both internal and external noise sources contribute to this burden. These results contribute to the limited evidence on environmental noise in schools in middle-income urban settings and support the inclusion of school acoustic environments within urban planning, building technologies and educational practices to create healthier acoustic environments, protecting teachers’ wellbeing.

**Supplementary Information:**

The online version contains supplementary material available at 10.1038/s41598-026-45322-6.

## Introduction

Noise is a growing environmental and public health challenge in urban settings, contributing to disease, reduced quality of life, and economic costs. It is estimated to cause tens of thousands of premature deaths annually and is associated with cardiovascular, metabolic, and neurological disorders, as well as mental health problems^1^. Beyond its direct health effects, noise undermines social wellbeing and sustainability by imposing hidden burdens on communities and health systems.

Non-auditory consequences of noise exposure include impaired learning and cognitive performance^2–4^, increased risk of diabetes and cardiovascular disease^5,6^, higher prevalence of depression and migraines^7,8^, and psychological outcomes such as anger, anxiety, and agitation^9,10^. Sleep disturbances are particularly common with nighttime noise exposure^11,12^ although it is conceivable that noise exposure during the day could have a carryover effect on sleep quality during the night^13,14^. Recognizing these risks, the World Health Organization recommends for instance that road traffic noise levels do not exceed 53 dB Lden and 45 dB Lnight^12^.

For teachers, exposure to high classroom noise has been linked to discomfort, vocal strain, stress, and increased risk of depression^15–19^. Noise exposure in school settings has also been associated with sleep disturbances, reduced wellbeing, and heightened annoyance, which may in turn impair occupational functioning and quality of life^20,21^. Important, the effects of noise on health vary depending on intensity, duration, source, and individual sensitivity^22^.

While occupational noise is traditionally studied in industrial environments and in relation to hearing outcomes, schools represent another important, though under-recognized, occupational and community setting^23^. For occupational settings, international regulations suggest action-limits around 80–85 dBA − 8 h for indoor noise^9^. Research has shown that indoor noise in schools can exceed 80 dBA, a level associated with adverse effects on both teachers and students^24^. Importantly, classroom noise levels relate with outdoor noise levels differently depending on for instance climate, window behavior, building characteristics and pedagogics. Furthermore, both internal and external noise sources can contribute to noise in the school environment, depending mainly on the activities carried out by students (internal) and the surroundings, especially in relation to traffic (external)^25^.

Despite the importance of this issue, data on school noise (both indoor and outdoor) remain scarce in low- and middle-income countries, including Brazil. While studies have documented classroom noise in other countries^26^, evidence from Brazil is limited to a few localized investigations reporting elevated sound pressure levels indoors and negative impacts on teachers’ health and wellbeing^15,27,28^. Regarding environmental noise levels in schools and other settings as well, researchers have noted the lack of high-quality environmental noise studies in developing countries, despite high population density, rapid urbanization and heavy traffic^20^.

São Paulo, one of the world’s largest metropolitan areas, combines intense road traffic and dense urban activity, conditions that exacerbate noise exposure overall and in schools as well. in and around schools. Primary public schools in Brazil are often built without specific acoustic design considerations, resulting in classrooms with poor sound insulation, high reverberation times, and ambient noise levels that exceed recommended limits, which can impair communication and learning processes. In many such schools, windows remain open throughout the year for ventilation, and minimal insulation or acoustic treatment is present to buffer outdoor noise, increasing the transmission of environmental sound into learning spaces^29^. Together, these structural, environmental and behavioural factors support the relevance of studying both outdoor and indoor noise levels in the school context and underscore the need for more comprehensive data in low- and middle-income countries like Brazil.

This study addresses this gap, bringing innovation and contributing to the advancement of knowledge in the field, as it characterizes continuous environmental noise in a sample of schools in western São Paulo, Brazil, integrating these results with teacher’s health data. Specifically, we aimed to (i) describe measured environmental noise levels, (ii) examine potential internal and external sources of noise exposure, and (iii) assess associations between noise levels and teachers’ health indicators. By providing evidence from a middle-income urban context like São Paulo, Brazil, where high-quality data on environmental noise are still scarce, our findings can inform strategies for noise surveillance and mitigation to support the development of healthier and more sustainable school environments.

## Materials and methods

We used a short-term repeated-measures design, with daily noise measurements (LAeq) over a school-week in seven schools in western São Paulo, to examine the association between potential noise sources and noise levels. For the analysis of teachers’ health outcomes, we applied a cross-sectional multilevel exposure design, in which daily noise exposure (LAeq, LAFmax) was recorded as described previously while health outcomes were assessed once through a standardized questionnaire. Schools were selected using data from the São Paulo Western Region Birth Cohort, which identified the schools where most cohort children were enrolled^30^. Of the ten eligible public primary schools invited by the Western Regional Board of Education, seven schools agreed to take part in the present study.

Of these seven schools, three units (schools 5, 6, and 7), which are for preschool, are located in educational complexes, divided into blocks according to the educational levels. The remaining schools are exclusively for preschool. According to municipal regulations, all early childhood education schools must ensure that activity rooms have at least 1.20 m² per child. In addition, the schools have other educational environments, such as courtyards, computer labs, and playgrounds. In the educational complexes, the areas reserved for preschool occupy separate physical spaces. In all of them, the classrooms are organized in blocks with circulation corridors (~ 2 m wide) between them, with access to or proximity to external activity areas. The larger schools (schools 6 and 7) have more classrooms for preschool (between 7 and 9 classrooms) and, therefore, a larger number of students. The others have 3 to 5 classrooms. The distribution of classrooms is, in general, linear. Thus, some teachers are located closer to the street and others a little further away. The average number of students was 170 children (Min: 60 children - School 2; Max: 350 children – School 5). Importantly, despite differences in size and proximity to surrounding noise sources, these schools lack acoustic or thermal insulation as well as air-conditioning systems.

All teachers at these seven schools were invited to participate. In total, 85 teachers completed the questionnaires, corresponding to a mean response rate of 65%. Data collection with teachers was conducted concurrently with the environmental noise measurements to ensure comparability between self-reported perceptions and objective exposure levels. The study protocol was approved by the Research Ethics Committee of the Hospital das Clinicas, Faculty of Medicine, University of São Paulo (CAAE: 43080920.5.0000.0068), as well as by the Western Regional Board of Education.

### Noise measurement

A-weighted equivalent sound pressure levels (LAeq) averaged at 1-second intervals continuously over one week were measured at these seven schools using a Type-II Sound Level Meter Data Logger Noise Sentry RT (Convergence Instruments, Sherbrooke, QC, Canada) (see figure S1 for an example of a week measurement for school X). They were previously calibrated to 94 dBA using a CR:514 calibrator (Cirrus Research plc). Adjustments were made when necessary, using dedicated software (http://convergenceinstruments.com/data-loggers-software/).

Outdoor measurements were taken from March 8 to 14, 2022; the meter placement criteria were street-level or the most heavily used location, as close as possible to the preschool classrooms. Meters were placed on rooftop structures and streetlights, always outdoors and facing the street (most exposed façade to surrounding urban activity), at a height of 2–3 m, and in locations away from pedestrian movement or handling. By focusing on a common external reference point, this method allows for meaningful comparisons between schools while avoiding variability related to classroom location, building layout, or individual movement patterns. The devices were removed one week after their installation, at the same time. The meter at one of the schools experienced an unexpected malfunction, preventing data from being recorded. Therefore, we used, for this school, noise data from another campaign, from October 6th to 12th, 2021, in which October 11th was a public holiday.

Based on these measurements, only the noise of interest for our research questions was selected for the analysis of potential noise sources and health effects, that is, from Monday to Friday, from 7 am to 7 pm (LAeq(7–19) and LAFmax(7–19)), which is the schools’ operating hours. The LAeq(7–19) is the equivalent continuous sound level representing the average exposure during school hours, and LAFmax(7–19) is the maximum sound level capturing short-term peak events during the same period.

The main exposure of interest was the effective environmental noise reaching the school façade, defined as the A-weighted sound level at the façade most exposed to traffic and surrounding community noise sources. This approach is consistent with international guidelines recommending the use of indicators such as Lden and Lnight at the most exposed façade for research on health effects^12^. The most exposed façade is commonly used in environmental noise epidemiology as a standardized proxy for the external acoustic load incident on a building, enabling comparability across study units and enhancing consistency in exposure assessment^6^. It is important to emphasize that our objective was not to estimate the individual indoor personal noise dose, but to focus on the shared contextual exposure by considering the school as the unit of exposure and assigning the measured noise level to all teachers within each school, in order to examine the differences between schools in noise environments and how their associations with differences in teachers’ health indicators.

### Data collection with teachers

A total of 85 teachers from the seven participating schools completed standardized questionnaires. The instruments were self-administered on paper, either filled out independently or with the support of school staff and took 20–30 min to complete. In this paper we focused on the following constructs and respective questionnaires:

**Noise sensitivity** was investigated using the NoiSeQ-R14 questionnaire^31^, to assess noise sensitivity at work (four questions; 0–12), during sleep (four questions; 0–12), at home (four questions; 0–12) and in general (one question; 0–3), using a 4-point verbal scale ranging from “strongly disagree” to “strongly agree” (score values from 0 to 3, respectively), resulting in a score range of 0–39. However, since the focus of this study is on the workplace noise, scores greater than or equal to 9 in the “work” category, as well as scores equal to 3 in the “general” category were classified as “highly sensitive” (HS).

**Noise annoyance** was assessed using ICBEN scale in relation to specific noise sources in the past 12 months. In this paper, we will focus on noise from road traffic, schools/playgrounds, and conversation/children’s screams, using an 11-point numerical scale where 0 means “not at all annoyed” and 10 means “extremely annoyed” ^32^. Scores between 8 and 10 for each noise source were classified as “highly annoyed” (HA).

**Self-rated health (SRH)** was based on the question “In general, how is your health?”. The answers were dichotomized into poor (fair, poor, and very poor) and good (good and very good).

**Sleep quality** was assessed based on four questions on distinct aspects of sleep: (i) difficulties falling asleep, (ii) agitated sleep, (iii) wake several times during the night and (iv) wake very early in the mornings. Answers could vary from 1 to 3 (never/rarely = 1, sometimes = 2, often/always = 3). We created a score with the sum of the answers for the four questions. This score could vary from 4 to 12, where 4 would mean better and 12 worse sleep quality.

**Mental health and wellbeing** were assessed based on five questions with three of them focused on negative perceptions of mental health (anxious, depressed, hopeless) and two on positive dimensions of mental health (relax, feel good). Answers could vary from 1 to 3 (never/rarely = 1, sometimes = 2, or often/always = 3). The positive dimensions were coded on an inverted scale where higher scores reflected never/rarely feeling relaxed or good. Then, we created a score with the sum of the answers for the five questions. This score could vary from 5 to 15, where 5 would mean better and 15 worse wellbeing.

### Other variables

*School level*.

We used school characteristics to explore potential noise sources at schools. These sources were classified into internal and external in relation to the schools. As an internal potential source, we included the number of students per school. External sources included characteristics related to road traffic (i.e., number of traffic lanes, presence of bus stop and of bus garage in the school surroundings), commercial activities (i.e., presence of restaurants, stores, bars and markets) and other type of activities (i.e., presence of parks or of other schools neighboring the schools).

*Individual level*.

We collected information on age, gender, and the number of working hours in the school.

## Statistical analysis

For the description of noise levels in the different schools, we present daily LAeq(7–19) and LAFmax(7–19). For the analysis on the potential noise sources at schools, we focused on weekdays, considering the school academic calendar. We compared noise levels across schools using linear regression models that included categorical indicators for schools to capture between school differences. To analyze potential noise sources, we fit separate linear regression models for each source at the school level, including categorical indicators for both school and day. This approach controlled for unobserved, time-invariant characteristics of schools and for daily variations common in school noise environments.

For the analysis of teachers’ health outcomes, we applied linear or logistic regression models depending on whether the health indicator was continuous or binary. Two models were estimated for each outcome: the first included schools as predictors, and the second replaced schools with average noise levels to assess whether noise exposure could explain the observed school effects, based on changes in R². Both models included categorical terms for day of measurement and were adjusted for age.

Although the data had a hierarchical structure, with exposure measured at the school level and outcomes assessed at the individual (teacher) level and teachers nested within schools, we did not apply multilevel random-effects models because reliable estimation of between-cluster variance requires an adequate number of higher-level units. When fewer than 10 clusters are available - as in our study, which included seven schools -variance components may be unstable, between-group variance underestimated, and standard errors biased^33^.

Instead, we used regression models with school fixed effects, an approach recommended when the number of clusters is limited and the primary interest lies in estimating associations within and between schools rather than partitioning variance across levels.34 In addition, the use of school-level fixed effects is also a way to correct for clustering, since it is unclear, for a small number of clusters, which method is more reliable. This approach controls for all observed and unobserved time-invariant characteristics at the school level (e.g., location, building features, surrounding environment) without requiring the estimation of random variance components or distribution assumptions for random effects^33–35^.

While increasing the number of individuals per school improves the precision of individual-level estimates, it does not compensate for an insufficient number of clusters when estimating contextual (between-school) variance^33^. Accordingly, clustering was addressed using school fixed effects, treating the school as the contextual unit of exposure. The sampling strategy included all eligible teachers within each participating school, thereby maximizing within-school representativeness and statistical power for analyses conducted at the individual level. A sensitivity analysis excluded data from one school and from teachers whose measurements overlapped with a previous campaign conducted during a holiday period. We report descriptive statistics, noise levels, regression coefficients, or odds ratios, and 95% confidence intervals. All analyses were performed using Stata version 18 (Stata Corp, College Station, TX, USA).

## Results

**Fig. 1:**
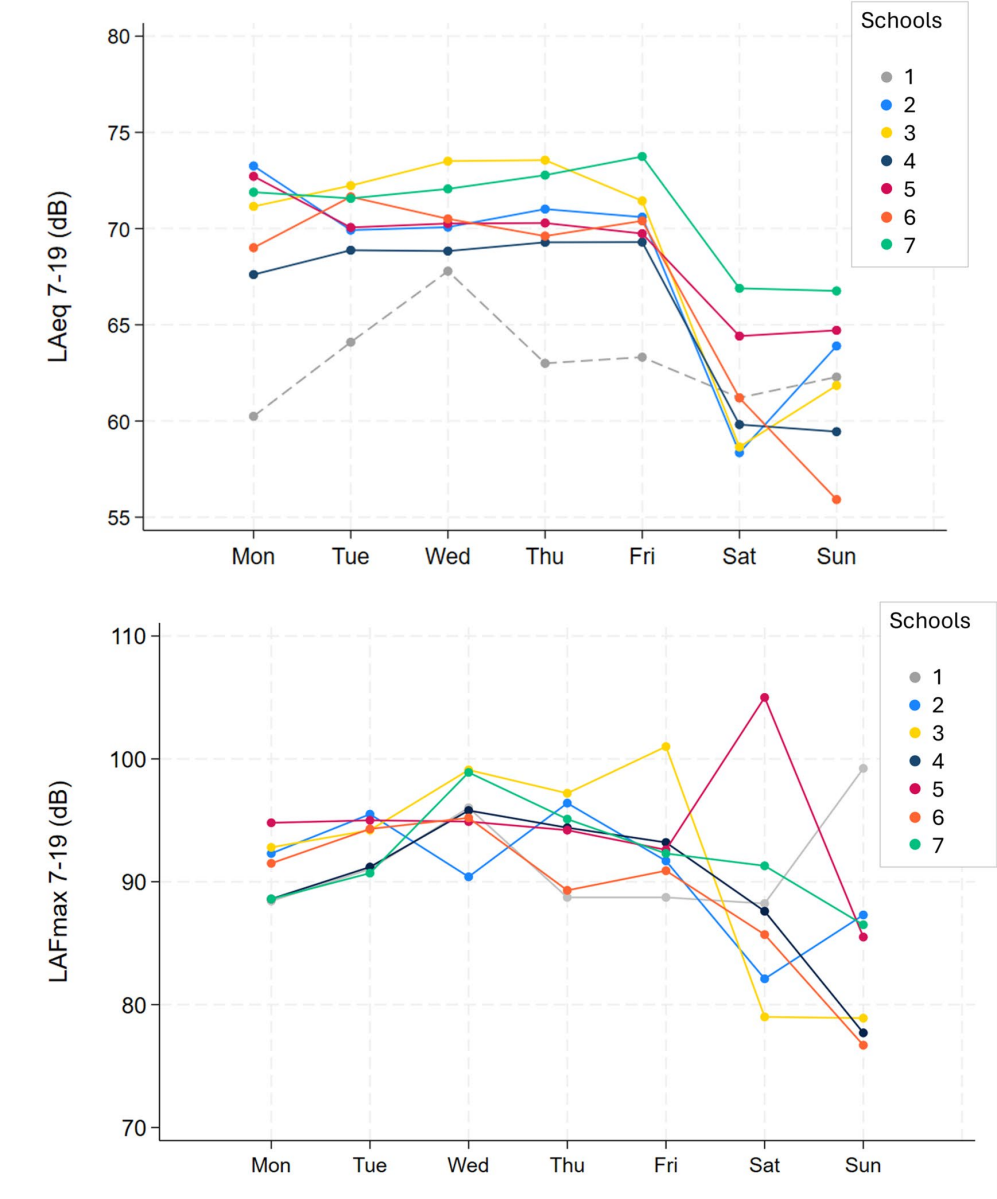
LAeq(7–19) and LAFmax(7–19) per day, per school

Overall, considering all schools and all days (including weekends), we report a median of 69.7 dB (IQR 64.1–71.1) LAeq(7–19) and 92.3 dB (IQR 88.6–95) LAFmax(7–19) in the same period of the day. If only focusing on schooldays, we report a median of 70.3 dB (IQR 69.4–71.9) LAeq(7–19) and 94.2 dB (IQR 90.9–95) LAFmax(7–19), characterizing higher exposure levels during schooldays. LAeq(7–19) and LAFmax(7–19) per day and per schools are reported in detail in the supplement (Table S1) and illustrated in Fig. 1. For most schools, school-days had higher LAeq(7–19) levels when compared to the weekends. We observed a consistent level of LAeq(7–19) per school during schooldays, except for school 1, which had measurements during a school holiday. For LAFmax(7–19), there was more variation in noise levels per school over the week and for the comparison between schooldays and weekends.

Table 1 shows schools’ characteristics that were considered as potential noise sources related to the sound environment at schools. These characteristics were classified as internal or external to the school: the number of students is the only indicator of internal sources whereas the external sources are related to noise from traffic, from commercial activities and/or from other activities (i.e., other schools and parks). Some schools were in areas with less traffic and commercial activities but surrounded by other schools and/or parks (i.e., schools 5 and 6). Among these, school 5 has the highest number of students (*n* = 350). Schools exposed to multiple traffic sources were also exposed to either commercial or other sources (e.g., 2, 3 and 4). Among these, school 2 had fewer students (*n* = 60). The distance between the outdoor noise monitoring location (most exposed façade) and the street varied markedly across the seven schools, ranging from 3 m to 145 m. This reflects relevant differences in school placement and surrounding urban layout, suggesting substantial heterogeneity in environmental noise contexts across schools.

**Table 1:** School characteristics concerning potential noise sources.

	School	1	2	3	4	5	6	7
Internal	Number of students	130	60	150	150	350	150	200
External related to traffic	Traffic lanes	3	4	4	4	3	2	2
	Crossings	No	Yes	Yes	Yes	No	No	Yes
	Bus stops	No	Yes	Yes	Yes	No	No	Yes
	Parking garage	Yes	No	No	No	Yes	No	No
	Overall ^a^	1	3	3	3	1	0	2
External commercial	Restaurants	Yes	No	Yes	No	No	No	No
	Stores	No	No	Yes	No	No	No	No
	Street markets	No	Yes	No	No	No	No	No
	Overall^b^	1	1	2	0	0	0	0
External others	Parks	No	Yes	No	No	Yes	Yes	Yes
	Schools	Yes	No	No	Yes	No	Yes	Yes
	Overall^c^	1	1	0	1	1	2	2
Distance (meters)	To the street	3	5	20	17	6	50	145
	From the ground	2.9	2.8	2.7	2.6	2.7	5.2	3

^a^ Overall exposure to external sources related to traffic (categorical, 0 to 3) meaning number of traffic sources (i.e., four traffic lanes, crossings within 100 m, bus stops, parking garage surrounding the schools).

^b^ Overall exposure to external sources related to commercial activities (categorical, 0 to 2) meaning number of commercial sources (i.e., restaurants, stores or street markets surrounding the schools).

^c^ Overall exposure to external sources related to others (categorical, 0 to 2) meaning number of other sources (i.e., parks and schools surrounding the schools).

Table 2 demonstrates the findings from the linear regression models on noise levels. Each potential predictor of school noise was included in a separate model. The exclusion of school number 1 did not affect the estimates but increased the model performance reflected by a higher R2. For the main results, we present the analysis including all school. Analysis excluding school 1 is on the Supplement (Table S2).

**Table 2:** Associations between noise levels during weekdays (LAeq(7–19) and LAFmax(7–19)) and potential noise sources.

	LAeq(7–19)			LAFmax(7–19)		
	dB change	95%CI		dB change	95%CI	
School						
1	-5.09	(-5.71;	-4.47)	-2.05	(-2.78;	-1.32)
2	2.19	(1.78;	2.60)	0.62	(-0.31;	1.55)
3	3.60	(3.26;	3.94)	4.22	(3.28;	5.16)
4	reference			reference		
5	1.83	(1.51;	2.16)	1.66	(1.03;	2.29)
6	1.45	(1.12;	1.79)	-0.4	(-1.13;	0.33)
7	3.63	(3.30;	3.96)	0.48	(-0.36;	1.32)
Number of students*	3.63	(3.30;	3.96)	0.48	(-0.36;	1.32)
Number of external noise source traffic related						
0	-0.38	(-0.70;	-0.06)	-2.06	(-2.59;	-1.53)
1	reference			reference		
2	1.80	(1.48;	2.11)	-1.18	(-1.86;	-0.50)
3	-1.83	(-2.16;	-1.51)	-1.66	(-2.29;	-1.03)
Number of external noise source commercial related						
0	-2.19	(-2.60;	-1.78)	-0.62	(-1.55;	0.31)
1	reference			reference		
2	1.41	(0.99;	1.82)	3.60	(2.55;	4.65)
External noise source parks and schools related						
0	3.60	(3.26;	3.94)	4.22	(3.28;	5.16)
1	reference			reference		
2	3.63	(3.30;	3.96)	0.48	(-0.36	1.32)

*Per 50 students. All models include fixed effect for school and day.

There were significant differences between schools concerning noise levels, markedly LAeq but not LAFmax. School 3 was exposed to consistently higher levels of LAeq(7–19) and LAFmax(7–19) while schools 1 and 4 were exposed to the lowest levels. Every increase in 50 students in a school (students per school ranging from 60 to 350) was associated on average with an increase in 3.6 dB LAeq(7–19). Regarding the external sources, the commercial ones had a clear trend with increasing noise levels following the increase in the number of exposure sources. For the traffic related noise sources, there was no clear trend. Analysis demonstrated an average increase of 1.8 dB in schools with two sources when compared to one traffic source while on the other hand schools surrounded by 3 sources, had an inverse association, with lower noise levels in comparison with schools with one traffic source (Table 2).

Sampled teachers were mostly female (92.9%), on average 44.4 years of age with the majority (75.3%) working 30 h or more per week. A significant proportion reported being highly annoyed by noise, especially from irrelevant conversations / screams (48.2%), road traffic noise (35%), and other schools (30.6%). More than 60% of teachers reported being highly sensitive to noise at work. Age group 40 to 50 years was the age group showing the highest proportion of individuals with negative health outcomes. There were no clear differences in the sample characteristics between the total sample and the sample excluding teachers from school 1 (Table 3).

**Table 3:** Teachers’ characteristics.

		Age groups			
	Total *N* = 85	< 40	40–50	> 50	School 1 excluded *N* = 73
Number of participant teachers per school, *n*					
School 1	12	2	8	2	
School 2	12	2	4	6	
School 3	10	5	5	0	
School 4	6	3	1	2	
School 5	21	7	6	8	
School 6	12	5	4	3	
School 7	12	2	6	4	
Female, n (%)	79 (92.9)	26 (96.3)	32 (87)	21 (84)	67(91.8)
Age (in years), mean ± SD	44.4 ± 7.84	35.5 ± 3.3	44.6 ± 3	53.9 ± 3	44.4 ± 8.15
Working hours ≥ 30 h,	64 (75.3)	17 (63)	28 (84.8)	19 (76)	56 (76.7)
Poor self-rated health, n (%) *	15 (17.9)	3 (11.1)	7 (21.9)	5 (20)	14 (19.4)
Wellbeing, mean ± SD*	9.8 ± 1.1	9.8 ± 1.1	9.9 ± 1.2	9.5 ± 1	9.7 ± 1.1
Sleep quality, mean ± SD*	8.1 ± 2.1	7.6 ± 2	8.4 ± 2.1	8.1 ± 2.3	8.1 ± 2.1
HA school, n (%)	26 (30.6)	5 (18.5)	13 (39.4)	8 (32)	23 (31.5)
HA conversation, n (%)	41 (48.2)	12 (44.4)	20 (60.6)	9 (36)	37 (50.7)
HA road, n (%)	30 (35.3)	11 (40.7)	11 (33.3)	8 (32)	26 (35.6)
Noise sensitivity, mean ± SD	27.7 ± 7.1	25 ± 6.5	30.4 ± 5.3	27 ± 8.6	28 ± 7.1
HS Work, n (%)	52 (61.2)	16 (59.3)	23 (69.7)	13 (52)	46 (63)
HS general, n (%)	32 (37.6)	5 (18.5)	19 (57.6)	8 (32)	28 (38.4)

*1 missing; HA, highly annoyed; HS, highly sensitive

For the teachers’ health indicators, we also performed the main analysis focusing on the entire sample and a sensitivity analysis a sample excluding teachers from school 1. We present the main findings from the main analysis in the manuscript and the sensitivity in the supplement (Table S1-S3). Based on main and sensitivity analysis, there were clear differences between the health of teachers in the different schools, markedly and consistently for noise sensitivity score, wellbeing, and sleep quality (Table 4). Schools explain some of the variations in the teachers’ health (R2 ranging from 0.02 to 0.13), markedly for HS in general, noise sensitivity score, poor SRH and HA from schools. LAeq(7–19) and LAFmax(7–19) were introduced in models 2a and 2b to evaluate whether the actual noise levels at schools could explain some of the variation observed between the schools and what could be the impact size of the association between noise levels and teachers’ health indicators. Fixed effects for schools were not used in models 2 because of the complete collinearity between schools and noise levels.

**Table 4:** Linear and logistic regression models on several indicators of teachers’ health according to different schools and adjusted for age. All schools included.

	Poor SRH	Wellbeing	Sleep quality	HA school	HA conversation	HA road	Noise sensitivity	HS work	HS general
	OR (95% CI)	β (95% CI)	β (95% CI)	OR (95% CI)	OR (95% CI)	OR (95% CI)	β (95% CI)	OR (95% CI)	OR (95% CI)
School									
1	0.44 (0.12; 1.64)	4.55 (1.44;7.67)	2.06 (0.75;3.37)	1.58 (0.47;5.23)	1.00 (0.39;2.53)	1.04 (0.41;2.59)	4.54 (-0.10;9.19)	1.01 (0.42; 2.46)	2.40 (1.00;1.05)
2	2.20 (0.70;6.91)	3.77 (0.92;6.61)	2.69 (1.45:3.92)	3.98 (1.23;12.81)	1.43 (0.57;3.59)	2.21 (0.88;5.51)	8.62 (3.84;13.47)	2.08 (0.84;5.19)	13.17 (4.18;41.52)
3	0.59 (0.16;2.17)	4.01 (1.52;6.62)	2.52 (1.25;3.79)	5.89 (1.81;19.18)	8.00 (2.85;22.47)	1.30 (0.50;3.34)	7.82 (3.31;12.34)	2.30 (0.90;5.92)	0.59 (0.15;2.31)
4	Reference	Reference	Reference	Reference	Reference	Reference	Reference	Reference	Reference
5	0.51 (0.16;1.58)	2.59 (0.18;4.99)	2.17 (0.96;3.39)	1.11 (0.35;3.45)	1.23 (0.52;2.89)	0.82 (0.35;1.93)	7.81 (3.39;12.2)	3.23 (1.38;7.56)	1.94 (0.67;5.64)
6	1.00 (0.31;3.23)	4.10 (0.23;7.96)	2.34 (0.84;3.83)	3.81 (1.20;12.11)	4.00 (1.58;10.15)	2.00 (0.80;4.97)	6.61 (1.94;11.28)	1.40 (0.58;3.39)	3.65 (1.20;11.09)
7	3.22 (1.07;9.67)	3.81 (0.83;6.80)	2.36 (1.06;3.67)	0.81 (0.24;2.77)	2.00 (0.80;4.99)	0.43 (0.16;1.19)	8.25 (3.54;12.95)	0.74 (0.30;1.81)	4.45 (1.47;13.50)
Age	1.02 (0.99;1.06)	0.05 (-0.08;0.19)	0.01 (-0.03;0.05)	1.05 (1.02;1.08)	1.00 (0.97;1.03)	0.98 (0.96;1.01)	0.10 (0.02;0.18)	0.99 (0.97;1.02)	1.03 (1.00;1.05)
R2	0.10	0.02	0.04	0.09	0.07	0.04	0.12	0.05	0.13

SRH, self-rated health; HA, highly annoyed; HS, highly sensitive. All models include a fixed term for time (days).

Noise indicators could not explain the variation in teachers’ general health indicators between schools, except for poor SRH. Every increase in 10dB in LAeq(7–19) was associated with an increase in 4.21 times higher odds of poor SRH, accounting for age (Table 5). In the analysis excluding teachers from school 1 (Table S4), we observed a tendency of similar effects concerning poor SRH, although the smaller sample size affected the precision of estimates. Interestingly, for sleep quality, with this restricted sample we observed an association between LAeq(7–19) levels in a way that every increase in 10dB was linked to an increase in 1.44 points in the sleep quality scale, reflecting worse sleep quality as noise exposure increases.

**Table 5:** Linear and logistic regression models on teachers’ general health, severe annoyance, and noise sensitivity indicators, according to LAeq(7–19) and LAFmax(7–19) levels. All schools included.

	LAeq(7–19)	LAFmax(7–19)	LAeq(7–19)	LAFmax(7–19)	LAeq(7–19)	LAFmax(7–19)
	Poor SRH		Mental wellbeing		Sleep quality	
	OR (95% CI)	OR (95% CI)	β (95% CI)	β (95% CI)	β (95% CI)	β (95% CI)
Noise level	4.21 (1.27;13.99)	1.00 (0.39;2.58)	-0.09 (-0.49;0.32)	0.01 (-0.38;0.40)	0.38 (-0.37;1.14)	0.27 (-0.47;1.02)
Age	1.05 (1.02; 1.08)	1.05 (1.02;1.08)	-0.00 (-0.02;0.01)	-0.00 (-0.02;0.01)	0.03 (0.00;0.05)	0.03 (0.00;0.05)
R2	0.04	0.02	0.00	0.00	0.01	0.00

	HA school		HA conversations / children’s screams		HA road traffic	
	OR (95% CI)	OR (95% CI)	OR (95% CI)	OR (95% CI)	OR (95% CI)	OR (95% CI)
Noise level	1.53 (0.73;3.22)	1.67 (0.74;3.75)	2.83 (1.41;5.67)	2.18 (1.07;4.41)	0.93 (0.49;1.76)	0.93 (0.44;1.96)
Age	1.04 (1.01;1.06)	1.04 (1.01;1.07)	0.99 (0.96;1.01)	0.99 (0.96;1.01)	0.98 (0.96;1.01)	0.98 (0.96;1.01)
R2	0.02	0.02	0.02	0.01	0.00	0.00

	Noise sensitivity		HS work		HS general	
	β (95% CI)	β (95% CI)	OR (95% CI)	OR (95% CI)	OR (95% CI)	OR (95% CI)
Noise level	4.16 (2.10;6.23)	2.59 (0.26;4.92)	1.57 (0.82;3.00)	2.11 (0.99;4.49)	1.30 (0.66;2.56)	0.52 (0.25;1.08)
Age	0.13 (0.05;0.21)	0.14 (0.06;0.22)	0.99 (0.96;1.01)	0.99 (0.97;1.01)	1.05 (1.03;1.08)	1.05 (1.03;1.08)
R2	0.05	0.03	0.00	0.01	0.03	0.03

SRH, self-rated health; HA, highly annoyed; HS, highly sensitive

*OR per 10dB increase in noise levels. All models include a fixed term for time (days)

For the noise annoyance analysis, every increase in 10dB LAeq(7–19) and LAFmax(7–19) was associated with higher odds of reporting HA from irrelevant conversations / screams of 2.83 and 2.18 times, respectively (see Table 5). The association between LAeq(7–19) and HA by conversation and screams was also demonstrated in the restricted sample, suggesting a more robust finding (see Table S4).

For noise sensitivity, every increase in 10dB in LAeq(7–19) and LAFmax(7–19) was linked to an increase in 4.16 and 2.59 points in the noise sensitivity scale, respectively. Findings concerning LAeq(7–19) and noise sensitivity score were also reported for the restricted sample analysis. For HS at work, there were borderline associations for the entire sample but not in the restricted sample (Table 5 and Table S4).

## Discussion

This study aimed to characterize noise levels in schools and to explore their association with teachers’ health and wellbeing. We observed that measured outdoor noise consistently exceeded recommended limits and that internal school factors, such as student numbers, and external school factors, including nearby traffic, commercial areas, and other schools or parks, influenced environmental noise exposure. Significant differences in teacher health indicators were also observed between schools, with higher noise levels associated with poor self-rated health, poorer sleep quality, and greater noise sensitivity. Teachers in their 40s and 50s appeared particularly vulnerable as they report higher rates of disadvantaged health.

Across the seven schools, median weekday outdoor noise levels exceeded WHO guidelines for road traffic noise^12^. Our findings indicate a level of exposure (70.3 dB LAeq(7–19) and 94.2 dB LAFmax(7–19)) potentially harmful to both teachers and students. Our results are consistent with prior research showing elevated school noise internationally, despite differences in methodologies. Hadzi-Nikolova, et al. ^26^ reported 58–63 dBA outside school buildings in Macedonia (measured over 2 years). In Malaysia, Ismail, et al. ^36^ observed 64–68 dBA in three schools (measured over 6 school- and non-school-days), with higher values on non-school days due to increased traffic, which contrasts with our findings. This could be because in Brazil school days coincide with peak traffic. Mamat, et al. ^25^ emphasized that traffic is the main contributing factor for the noise present in schools. Between country comparisons highlight the need to account for contextual and urban factors. As noted by Morillas, et al. ^37^, local characteristics such as population size, road use, and street design strongly influence practices and thus the effective environmental noise reaching the school façade.

In our study, each increase of 50 students was associated with an average 3.63 dB rise in LAeq(7–19), confirming prior evidence that students are one of the major sources of school noise^25,38^. This finding underscores the importance of educational interventions aimed at promoting quieter behavior in classrooms. Regarding external factors, the presence of commercial establishments showed a clear trend of increased noise, consistent with Ismail, et al. ^36^, who found higher exposure in schools near industrial and commercial areas, likely due to greater traffic and heavy vehicles in the latter.

Traffic-related noise indicators showed no consistent pattern in our analysis. While schools with one traffic source had higher exposure, those with three sources had lower levels, suggesting that other traffic, structural and environmental characteristics (e.g., distance from the meters to the roads, amount of traffic, building layout, vegetation, or isolation of the school site) may moderate exposure. When we analyzed the LAeq(7–19) by school, we found that the variability of noise levels over the week is low (especially considering weekdays), indicating that each of them has its own characteristics, suggesting that our hypothesis is credible. Parks and green spaces appeared protective in some cases, as confirmed by Feng, et al. ^39^, who reported noise reduction benefits of urban vegetation. However, when parks or other schools generate additional activity (which is conceivable), noise exposure might increase, indicating a complex interplay between environmental features, human activity, and noise.

Regarding teacher outcomes, our results revealed significant differences between schools in noise sensitivity, wellbeing, and sleep quality. Based on descriptive analysis, teachers aged 40–50 years presented worse health indicators which is consistent with some studies^20,21^, though the literature is mixed, with other research showing greater impacts among younger individuals^40,41^. In our analysis, because of sample size limitations we have addressed the different age groups although analysis was adjusted for age. Importantly, differences across studies may reflect sample characteristics, types of noise sources, and measurement methods^42^ as well as statistical modelling. Age has been cited as one of the factors that can influence noise sensitivity and, therefore, may influence greater noise annoyance and noise-related health problems. Some studies have found that younger individuals reported more noise-related health problems or impacts^40,41^. Conversely, other research suggests that older adults had a higher prevalence of noise-related health problems or annoyance^20^ or greater sensitivity to noise as age increases. In our study, we found that health indicators were poorer overall among the 40–50 age group. However, it should be noted that conflicting findings across studies may be related to the different characteristics of the samples and noise sources evaluated, the instruments used, and other variables that influence noise sensitivity in addition to age^42^.

Noise annoyance was frequent, particularly from children’s irrelevant conversations / shouting (~ 48%), road traffic (~ 35%), and neighboring schools (~ 30%). Similar findings were reported by Enmarker and Boman^43^, where 58% of teachers were most disturbed by irrelevant conversations, and by Rezende, et al. ^15^, who found that children’s indiscipline was the most disturbing classroom factor. It is known that any unwanted sound is considered noise, which can cause annoyance, as it impairs communication and activities, potentially generating feelings of irritation, discomfort, anguish, or frustration when the noise interferes with thoughts, feelings, or ongoing activities^43^. From this perspective, in the case of the school environment, irrelevant conversations/shouting from children, inside or outside the classroom, can cause annoyance to teachers, as they can negatively impact the activities they carry out.

Due to the low prevalence of males in our sample, we did not analyze the effect of gender on the perception of noise annoyance, although Enmarker and Boman^43^ found no differences and the findings are inconsistent on this issue. Annoyance prevalence was higher in our study as well as in South Africa when compared to estimates in European populations^44^. Some of the observed differences may be related to the different contexts in which the studies were developed, the number of individuals evaluated, and methodologies, as well as sociodemographic differences. Furthermore, factors such as attitude toward the noise source, personality, and noise sensitivity may interfere to a greater or lesser extent^45,46^. While most prior work has focused on exposure–response relationships for transport noise, we found no earlier studies linking increased school noise levels (either indoor or outdoor) to higher odds of teachers being highly annoyed by children’s irrelevant conversations / shouting.

In the present study, outdoor noise indicators failed to explain the variation in teachers’ general health indicators across schools, except for poor self-rated health. Each 10 dB increase in LAeq(7–19) was linked to a more than fourfold higher odds of poor self-rated health, echoing prior findings of associations between transportation noise (i.e., aircraft and road traffic noise) and health^47^. Sleep quality was also negatively affected, with higher noise associated with poorer outcomes, in line with evidence from systematic reviews^14^ and studies on teachers^43^.

In our study, more than 60% of teachers were highly sensitive to noise at work, and 37% in general. Sensitivity is a stable personal trait influencing reactions to environmental noise and has been linked to poorer quality of life^45,46^. Shepherd, et al. ^46^ have also used the NoiseQ questionnaire to measure environmental noise sensitivity in New Zealand adults. The authors found that for all categories assessed, as well as for overall noise sensitivity, 51% of the sample had scores indicating greater noise sensitivity. Our findings align also with Sieber, et al. ^44^, who observed higher percentages of highly noise-sensitive individuals in South Africa compared to Switzerland for the “general” category (women: 35.1% vs. 26.9%; men: 25% vs. 20.5%) and “work” (women: 36% vs. 26.5%; men: 32.1% vs. 23.1%) categories. The authors mentioned that variables such as sociodemographic factors, housing, traffic, and vulnerability could influence the perception of noise sensitivity. Furthermore, scale cutoff points, scales used, and sample sizes can influence the results observed in different countries, potentially explaining the difference between our findings and those of previous studies. Evidence indicates that noise sensitivity reflects both acoustic factors, such as exposure levels, and non-acoustic ones, including personality, attitudes, and prior experiences. This multidimensionality helps explain variability across studies and highlights noise sensitivity as a key factor shaping teachers’ health responses to environmental noise^48^.

Noise sensitivity, shaped by physiological, psychological, and lifestyle factors, heightens reactions to noise and is closely linked to stress and annoyance^23,46^. Evidence shows it also affects teachers’ physical and mental health, underscoring the need for preventive measures in schools. The SAPALDIA study confirmed strong associations between noise sensitivity, annoyance, and reduced quality of life^49^. In our study, higher exposure was tied to poorer sleep quality, echoing reviews that highlight sleep disorders as a common non-auditory effect of noise among school staff^14,43^. Each 10 dB increase further raised the odds of teachers being highly annoyed by children’s irrelevant conversations / shouting, a frequently reported source of disruption in schools^15,19,24^. These results point to interconnected pathways where noise sensitivity amplifies annoyance, sleep disturbance, and stress, undermining teachers’ health.

This study has several methodological considerations that merit discussion. Excluding School 1 slightly improved model performance but did not materially alter the results, supporting its inclusion in the main analyses. Marked differences in noise exposure were observed between schools, with School 3 exhibiting the highest levels and Schools 1 and 4 the lowest levels. However, since our main exposure contrast of interest was between schools, and not within schools, and the measured LAeq(7–19) levels were consistent within each school across the days of the week, we can suggest that the acoustic environments were stable at the school level. Also, the teacher sample was predominantly female, with a mean age of 44 years, and most participants worked 30 h or more per week, reflecting the typical profile of public-school teachers in Brazil.

Additionally, the participating schools lacked acoustic insulation and relied on natural ventilation, facilitating the noise transmission of external and internal sources between the different areas. The observed association between the number of students and LAeq (7–19) further suggests that school size contributes to internal noise levels. Taken together, these factors support the conceptualization of noise as a shared contextual exposure at the school level, consistent with approaches used in environmental noise epidemiology studies^50^.

The study also has important limitations. The relatively small sample size and gender imbalance may have reduced statistical power. Noise data for one school were obtained from a separate measurement campaign, although sensitivity analyses suggested that this did not influence the overall findings. All teachers within the same school were assigned a common exposure metric since indoor or individual-level noise measurements were not performed. Consequently, potential intra-school variation in exposure (e.g., due to classroom location or façade orientation) could not be directly quantified. Such variation would most likely result in non-differential exposure misclassification, as already mentioned^51^. Future studies should incorporate indoor and/or personal noise monitoring to refine exposure characterization and to further disentangle contextual and individual components of noise exposure in school settings.

Although the use of personal or wearable noise monitoring devices could provide more detailed individual exposure data, they also pose methodological challenges in school settings, including compliance issues, movement-related artefacts, and contamination by teachers’ own voice. In addition, the small number of participating schools precluded the use of multilevel random-effects models, and school fixed-effects regression was therefore applied, which controls for time-invariant school characteristics but limits generalizability beyond the sampled schools. The relatively small number of teachers within each school further constrained statistical power for detecting within-school associations and limited the precision of estimates, particularly for less prevalent health outcomes. Finally, the lack of information on other individual-level factors, such as teaching subject, professional experience, commuting time, or classroom acoustics, means that residual confounding cannot be excluded.

Future research should combine outdoor and indoor noise measurements, incorporate individual-level exposure assessment, and account for occupational, contextual and behavioural factors such as teaching experience, administrative duties, and commuting patterns. Studies including schools from different regions and educational contexts would also improve representativeness, as the schools in the present study were selected from the catchment area of a primary cohort. Despite these limitations, the study has notable strengths, including continuous week-long noise monitoring (capturing outdoor and indoor noise sources), the integration of objective environmental measurements with teachers’ health data, and the generation of evidence from a middle-income urban context where such data remain scarce. As such, these findings contribute to the growing evidence base on environmental noise in schools and have potential implications for public policy and urban and school planning aimed at mitigating harmful noise exposure.

## Conclusion

Our findings indicate that environmental noise levels measured at the most exposed façades of the seven participating schools exceeded recommended guidelines, with a median of 70.3 dB LAeq (7–19). These levels reflect substantial variability in the acoustic environments surrounding schools in this urban context. Factors such as the number of students and the presence of nearby commercial areas were associated with higher noise levels. At the teacher level, higher noise levels were associated with poor self-rated health and sleep quality, higher odds of reporting severe annoyance, and noise sensitivity. These findings suggest that both internal and external factors contribute to the overall sound environment and, although the exposure metric reflects contextual environmental conditions rather than individual indoor dose, the observed associations indicate that sustained school-level noise environments may have implications for teachers’ wellbeing. In doing so, the study contributes to the limited evidence on environmental noise in schools in middle-income urban settings and support consideration of school acoustic environments within policies in school and urban planning to create healthier acoustic environments, protecting teachers’ wellbeing.

## Supplementary Information

Below is the link to the electronic supplementary material.

Supplementary Material 1

## Data Availability

The datasets generated and/or analyzed during the current study are not publicly available due to the sensitive nature of the data but are available from the corresponding author on reasonable request and with appropriate ethical approval.
