# Supplementary material for: Environmental noise exposure in schools in São Paulo, Brazil: potential noise sources and health impacts among teachers
Source: Sci Rep. 2026 Mar 26;16:9979. doi: 10.1038/s41598-026-45322-6 (PMC13022274; doi:10.1038/s41598-026-45322-6)
Supplement: Supplementary file 1 — Supplementary Material 1 [file 41598_2026_45322_MOESM1_ESM.docx]

# Supplement


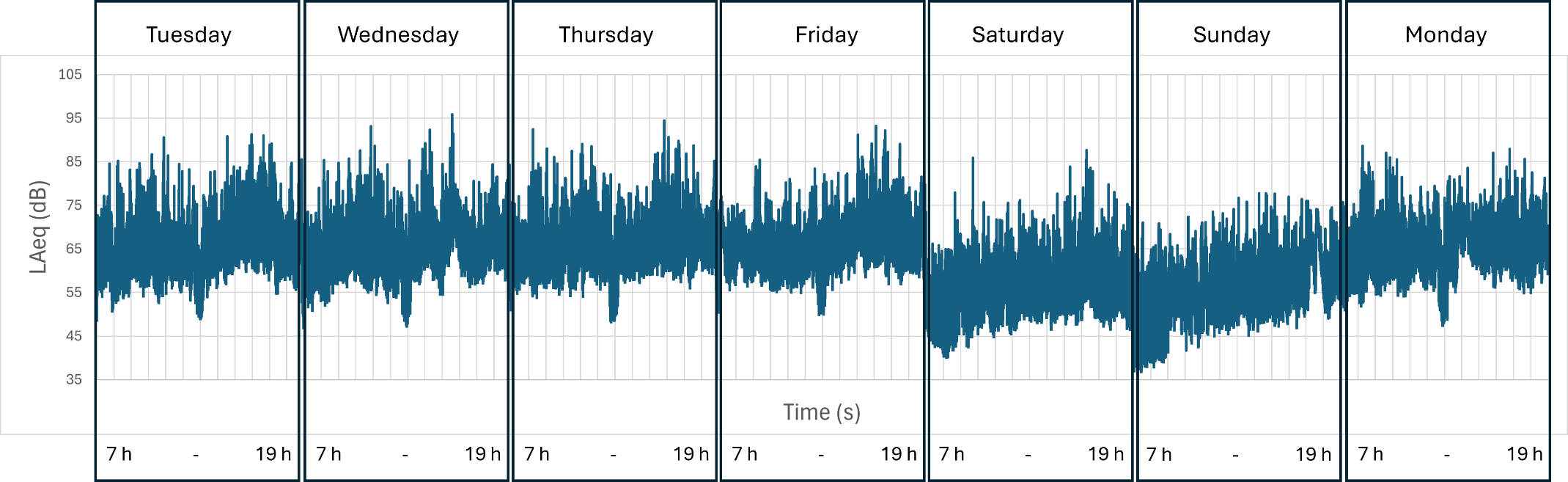


Figure S1. Example from one school (School 4) noise measurements (LAeq) per second for the period from 7h to 19h daily for a week, including the weekend. These values were used for the calculation of equivalent levels per day used in the analysis.

Table S1. LAeq (7-19) and LAFmax(7-19) per day, per school.

|  | **Weekdays** | | | | | **Weekend** | |  |
| --- | --- | --- | --- | --- | --- | --- | --- | --- |
| **School** | **1** | **2** | **3** | **4** | **5** | **6** | **7** | **Median (IQR)** |
|  | **LAeq(7-19)** | | | | | | | |
| 1* | 60.2 | 64.1 | 67.8 | 63 | 63.3 | 61.2 | 62.3 | 63 (61.2-64.1) |
| 2 | 73.2 | 69.9 | 70.1 | 71.0 | 70.6 | 58.3 | 63.9 | 70.1 (63.9-71) |
| 3 | 71.1 | 72.2 | 73.5 | 73.6 | 71.4 | 58.6 | 61.8 | 71.4 (61.8-73.5) |
| 4 | 67.6 | 68.9 | 68.8 | 69.3 | 69.3 | 59.8 | 59.4 | 68.8 (59.8-69.3) |
| 5 | 72.7 | 70.1 | 70.3 | 70.3 | 69.7 | 64.4 | 64.7 | 70.1 (64.7-70.3) |
| 6 | 69.01 | 71.6 | 70.5 | 69.6 | 70.4 | 61.2 | 55.9 | 69.6 (61.2-70.5) |
| 7 | 71.9 | 71.6 | 72.1 | 72.8 | 73.7 | 68.9 | 66.8 | 71.9 (66.9-72.8) |
|  | **LAFmax (7-19)** | | | | | | | |
| 1* | 88.4 | 91 | 96 | 88.7 | 88.7 | 88.2 | 99.2 | 88.7 (88.4-96) |
| 2 | 92.3 | 95.5 | 90.4 | 96.4 | 91.7 | 82.1 | 87.3 | 91.7 (87.3-95.5) |
| 3 | 92.8 | 94.2 | 99.1 | 97.2 | 101 | 79 | 78.9 | 94.2 (79-99.1) |
| 4 | 88.6 | 91.2 | 95.8 | 94.4 | 93.2 | 87.6 | 77.7 | 91.2 (87.6-94.4) |
| 5 | 94.8 | 95 | 94.9 | 94.2 | 92.6 | 105 | 85.5 | 94.8 (92.6-95) |
| 6 | 91.5 | 94.3 | 95.2 | 89.3 | 90.9 | 85.7 | 76.7 | 90.9 (85.7-94.3) |
| 7 | 88.6 | 90.7 | 98.9 | 95.1 | 92.3 | 91.3 | 86.5 | (88.6-95.1) |

*Measurements in another campaign, from October 6 to 12, 2021, with October 11 being a public holiday.

Table S2. Associations between noise levels during weekdays (LAeq(7-19) and LAmax(7-19)) and school characteristics (School 1 excluded).

|  | **LAeq(7-19)** | | | **LAFmax(7-19)** | | |
| --- | --- | --- | --- | --- | --- | --- |
|  | **dB change** | **95%CI** | | **dB change** | **95%CI** | |
| School |  |  |  |  |  |  |
| 2 | 2.19 | (1.80; | 2.58) | 0.62 | (-0.26; | 1.50) |
| 3 | 3.60 | (3.17; | 4.03) | 4.22 | (3.29; | 5.15) |
| 4 | *reference* | | | *reference* | | |
| 5 | 1.83 | (1.49; | 2.17) | 1.66 | (1.04; | 2.28) |
| 6 | 1.45 | (1.04; | 1.87) | -0.4 | (-1.16; | 0.36) |
| 7 | 3.63 | (3.26; | 4.00) | 0.48 | (-0.39; | 1.35) |
| Number of students* | 3.63 | (3.26; | 4.00) | 0.48 | (-0.39; | 1.35) |
| External sources traffic related |  |  |  |  |  |  |
| 0 | -0.38 | (-0.71; | -0.05) | -2.06 | (-2.63; | -1.49) |
| 1 | *reference* | | | *reference* | | |
| 2 | 1.80 | (1.52; | 2.07) | -1.18 | (-1.88; | -0.48) |
| 3 | -1.83 | (-2.17; | -1.49) | -1.66 | (-2.28; | -1.04) |
| External sources commercial related |  |  |  |  |  |  |
| 0 | -2.19 | (-2.58; | -1.80) | -0.62 | (-1.50; | 0.26) |
| 1 | *reference* | | | *reference* | | |
| 2 | 1.41 | (1.01; | 1.81) | 3.60 | (2.60; | 4.60) |
| External sources parks and schools |  |  |  |  |  |  |
| 0 | 3.60 | (3.17; | 4.03) | 4.22 | (3.29; | 5.15) |
| 1 | *reference* | | | *reference* | | |
| 2 | 3.63 | (3.26; | 4.00) | 0.48 | (-0.39 | 1.35) |

*Per 50 students. All models include fixed effect for school and day.

Table S3. Linear and logistic regression models on several indicators of teachers’ health according to different schools and adjusted for age. School 1 excluded.

|  | **Poor SRH** | **Wellbeing** | **Sleep quality** | **HA school** | **HA conversation** | **HA road** | **Noise sensitivity** | **HS work** | **HS gene** |
| --- | --- | --- | --- | --- | --- | --- | --- | --- | --- |
|  | **OR**  **(95% CI)** | **β (95% CI)** | **β (95% CI)** | **OR**  **(95% CI)** | **OR (95% CI)** | **OR**  **(95% CI)** | **β (95% CI)** | **OR**  **(95% CI)** | **OR**  **(95% CI)** |
| Schools |  |  |  |  |  |  |  |  |  |
| 2 | 2.12 (0.67;6.73) | 0.68 (0.28;1.08) | 2.05 (1.17:2.93) | 3.82 (1.13;12.91) | 1.39 (0.55;3.51) | 1.98 (0.79;4.96) | 8.63 (3.79;13.46) | 2.03 (0.82;5.04) | 13.19 (4.19;41.52) |
| 3 | 0.60 (0.16;2.21) | 0.54 (0.17;0.90) | 2.13 (1.25;3.02) | 6.32 (1.85;21.53) | 8.07 (2.86;22.77) | 1.34 (0.51;3.48) | 7.83 (3.31;12.36) | 2.32 (0.91;5.95) | 0.59 (0.15;2.31) |
| 4 | *Reference* | *Reference* | *Reference* | *Reference* | *Reference* | *Reference* | *Reference* | *Reference* | *Reference* |
| 5 | 0.50 (0.16;1.56) | 0.40 (0.07;0.74) | 1.62 (0.80;2.44) | 1.09 (0.34;3.55) | 1.22 (0.52;2.89) | 0.80 (0.33;1.91) | 7.80 (3.37;12.23) | 3.21 (1.38;7.48) | 1.94 (0.67;5.64) |
| 6 | 0.50 (0.13;1.89) | 0.10 (-0.33;0.52) | 2.26 (1.38;3.15) | 3.96 (1.18;13.21) | 4.01 (1.57;10.21) | 2.00 (0.80;5.00) | 6.61 (1.93;11.29) | 1.40 (0.58;3.39) | 3.65 (1.20;11.09) |
| 7 | 3.14 (1.05;9.42) | 0.67 (0.26;1.07) | 1.50 (0.64;2.35) | 0.78 (0.22;2.75) | 1.96 (0.78;4.90) | 0.40 (0.14;1.09) | 8.22 (3.49;12.95) | 0.72 (0.30;1.77) | 4.46 (1.47;13.51) |
| Age | 1.03 (0.99;1.07) | -0.02  (-0.03; -0.00) | 0.03  (0.01;0.06) | 1.07 (1.03;1.10) | 1.00 (0.98;1.03) | 1.00 (0.97;1.03) | 0.11 (0.02;0.19) | 1.00 (0.97;1.02) | 1.03 (1.00;1.06) |
| *R2* | 0.11 | 0.05 | 0.10 | 0.12 | 0.07 | 0.05 | 0.12 | 0.05 | 0.14 |

SRH= self-rated health; HA= highly annoyed; HS= highly sensitive. All models include a fixed term for time (days)

Table S4. Linear and logistic regression models on teachers’ general health, severe annoyance and noise sensitivity indicators, according to LAeq(7-19) and LAFmax(7-19) levels. School 1 excluded.

|  | **LAeq** | **LAmax** | **LAeq** | **LAmax** | **LAeq** | **LAmax** |
| --- | --- | --- | --- | --- | --- | --- |
|  | ***Poor SRH*** | | ***No wellbeing*** | | ***Sleep quality*** | |
|  | **OR (95% CI)** | **OR (95% CI)** | **β (95% CI)** | **β (95% CI)** | **β (95% CI)** | **β (95% CI)** |
| Noise level | 7.21 (1.07;48.7) | 0.60 (0.19;1.85) | 0.93 (0.21;1.66) | 0.19 (-0.24;0.62) | 1.44 (-0.07;2.94) | 0.30 (-0.52;1.11) |
| Age | 1.06 (1.02;1.09) | 1.05 (1.02;1.08) | -0.01 (-0.02;0.00) | -0.01 (-0.02;0.00) | 0.03 (0.01;0.06) | 0.03 (-0.01;0.06) |
| R2 | 0.04 | 0.03 | 0.02 | 0.01 | 0.03 | 0.02 |
|  | ***HA school*** | | ***HA conversation/screams*** | | ***HA road*** | |
|  | **OR (95% CI)** | **OR (95% CI)** | **OR (95% CI)** | **OR (95% CI)** | **OR (95% CI)** | **OR (95% CI)** |
| Noise level | 1.73 (0.36;8.41) | 1.63 (0.64;4.15) | 5.06 (1.17;21.8) | 1.71 (0.76;3.82) | 0.40 (0.09;1.82) | 0.88 (0.37;2.08) |
| Age | 1.05 (1.02;1.07) | 1.05 (1.02;1.08) | 0.99 (0.96;1.01) | 0.99 (0.97;1.02) | 1.00 (0.97;1.03) | 1.00 (0.97;1.02) |
| R2 | 0.02 | 0.02 | 0.01 | 0.00 | 0.00 | 0.00 |
|  | ***Noise sensitivity*** | | ***HS work*** | | ***HS general*** | |
|  | **β (95% CI)** | **β (95% CI)** | **OR (95% CI)** | **OR (95% CI)** | **OR (95% CI)** | **OR (95% CI)** |
| Noise level | 8.09 (2.13;14.05) | 1.57 (-1.19;4.33) | 0.65 (0.14;3.06) | 1.84 (0.77;4.40) | 1.29 (0.29;5.65) | 0.36 (0.15;0.84) |
| Age | 0.13 (0.05;0.22) | 0.15 (0.06;0.23) | 0.99 (0.97;1.02) | 0.99 (0.97;1.02) | 1.05 (1.03;1.08) | 1.05 (1.03;1.08) |
| R2 | 0.05 | 0.03 | 0.00 | 0.00 | 0.03 | 0.04 |

SRH= self-rated health; HA= highly annoyed; HS= highly sensitive. *OR per 10dB increase in noise level. All models include a fixed term for time (days).
